# Short-term outcomes after hybrid unicompartmental knee arthroplasty: A retrospective cohort study with minimum 3-year follow-up

**DOI:** 10.1097/MD.0000000000047572

**Published:** 2026-03-20

**Authors:** Yunchao Zhao, Tingting Zhou, Qingpeng Fang, Yijie Yang, Pengpeng Wang, Xiaoming Li, Jianyong Zhao

**Affiliations:** a Department of Traditional Chinese Orthopedics, Graduate School, Hebei University of Chinese Medicine, Shijiazhuang, Hebei Province, China; b Department of Orthopedics, Cangzhou Hospital of Integrated Traditional Chinese Medicine and Western Medicine of Hebei Province, Cangzhou, Hebei Province, China; c Department of Hebei Key Laboratory of Integrated Traditional Chinese and Western Medicine in Osteoarthrosis Research, Cangzhou Hospital of Integrated Traditional Chinese Medicine and Western Medicine of Hebei Province, Cangzhou, Hebei Province, China; d Department of Anesthesiology, Cangzhou Hospital of Integrated Traditional Chinese Medicine and Western Medicine of Hebei Province, Cangzhou, Hebei Province, China; e Department of Nursing, Cangzhou Hospital of Integrated Traditional Chinese Medicine and Western Medicine of Hebei Province, Cangzhou, Hebei Province, China.

**Keywords:** follow-up, hybrid, Oxford phase-3, retrospective study, short-term, survival rate, unicompartmental knee arthroplasty

## Abstract

This study aims to evaluate the survival rate and short-term clinical outcomes of hybrid unicompartmental knee arthroplasty (UKA). We retrospectively analyzed 155 Oxford phase-3 hybrid UKAs in 155 patients who were followed for more than 3 years. Kaplan–Meier survival curves were generated using revision as an endpoint. Oxford knee score (OKS) and range of motion (ROM) were evaluated for clinical assessment, and radiographs were used to assess implant-related complications. At an average follow-up of 3.6 years (range, 3–6 years), 2 knees required revision. The reasons for revision were bearing dislocation and unexplained pain. The overall survival rate was 98.7% at the 6-year follow-up. The mean OKS decreased from 40.94 ± 4.86 to 14.84 ± 1.39 at the last follow-up (*P* < .001). The mean OKS showed a significant improvement during the first 2 years. The mean ROM improved from 104.81 ± 10.03° to 114.93 ± 7.51° at the last follow-up (*P* < .001). The mean ROM increased during the first 3 years. Radiolucent lines were observed in 6 cases at 6 years postoperatively, affecting 4 knees. At 6 years postoperatively, the following complication rates were observed: overall revision rate: 1.33% (2/150); deep vein thrombosis rate: 3.33% (5/150); chronic soft tissue pain rate: 1.33% (2/150); stiffness rate: 0.67% (1/150); prosthesis dislocation rate: 0.67% (1/150); lateral meniscus injury rate: 0.67% (1/150); lateral arthritis progression rate: 2.67% (4/150). Oxford phase-3 hybrid UKA provides good survival rates and clinical outcomes in the short-term follow-up.

## 1. Introduction

Unicompartmental knee arthroplasty (UKA) for the treatment of knee anteromedial osteoarthritis has shown good clinical outcomes.^[1]^ However, there are reports indicating that postoperative prosthesis loosening, dislocation, reoperation, and revision rates are relatively high.^[2-5]^ Based on the fixation method, cemented UKA^[6]^ and cementless UKA^[7]^ are commonly used to treat knee anteromedial osteoarthritis, whereas hybrid UKA is less commonly used in clinical practice. Cemented prostheses provide initial stability, and cemented UKA offers more advantages over cementless UKA, particularly for the tibial plateau.^[8]^ Bone loss in cemented UKA during revision is greater than that in cementless UKA. Additionally, small tibial plateau prostheses are more likely to cause fractures in cementless UKA than in cemented UKA.^[3,9]^ Surgeons should be aware that excessive interference fit in cementless UKAs, combined with an impaction technique, may increase the risk of periprosthetic tibial fractures compared to cemented UKAs. Therefore, cementless UKA is less forgiving of surgical errors when inserting the tibial plateau component.^[10]^ The cementless femoral component has a lower occurrence of radiolucent lines (RLLs) than cemented prostheses and results in less bone loss during revision.^[11-13]^ We prefer to choose hybrid UKA over cemented UKA for shorter patients with normal or decreased bone mineral density (BMD). However, the short-term survival and clinical outcomes of hybrid UKA remain unknown. Recent studies have begun to explore the benefits of hybrid UKA, particularly in terms of combining the advantages of both cemented and cementless fixation methods. Studies comparing different fixation strategies in joint arthroplasty have reported broadly comparable clinical outcomes between hybrid and cemented fixation approaches,^[12–14]^ suggesting that hybrid fixation may also be a feasible strategy in UKA. These studies provide growing evidence that hybrid UKA could be an effective alternative for patients with specific anatomical and clinical characteristics, such as those with low BMD or shorter tibial dimensions. We hypothesized that hybrid UKA would demonstrate favorable short-term outcomes in a specific patient demographic. The purpose of our study was to evaluate the survivorship, clinical, and radiographic outcomes of Oxford phase-3 hybrid UKA during a 3.6-year follow-up (range, 3–6 years).

## 2. Materials and methods

### 2.1. Materials

**Figure 1.**
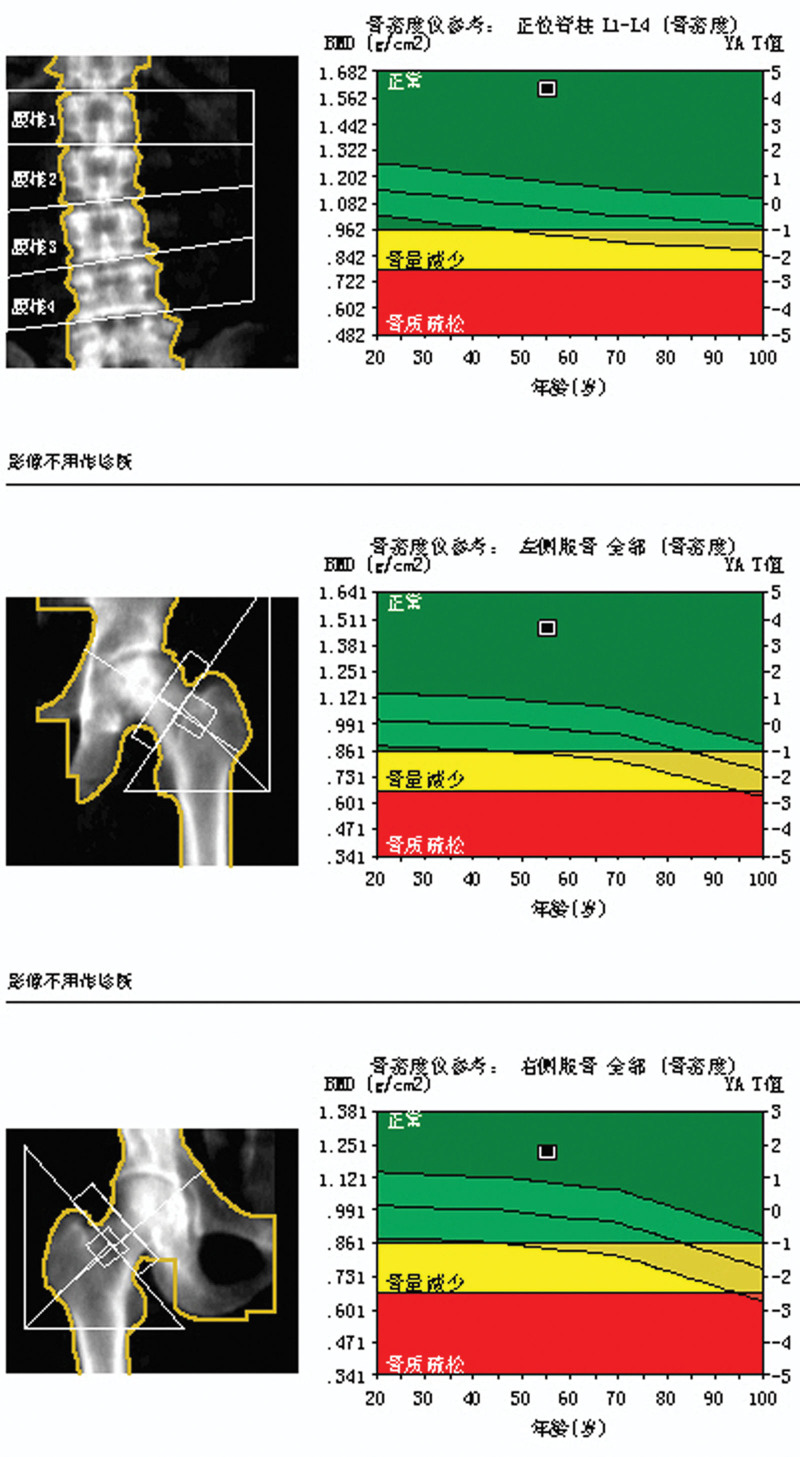
Normal BMD of 1 case. Bone mineral density (BMD) is considered normal when the measured values fall within the established normal reference range. BMD = bone mineral density.

**Figure 2.**
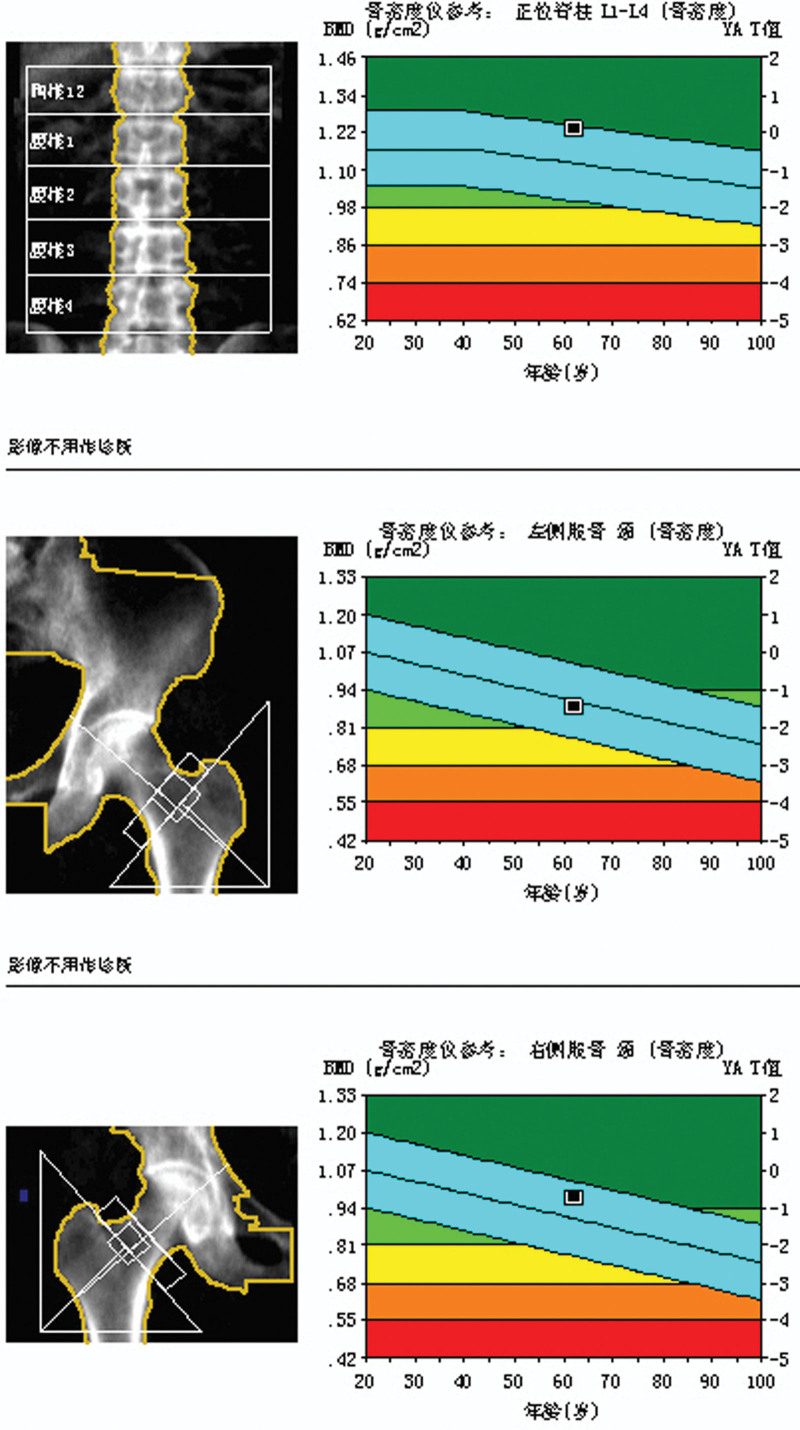
decreased BMD of 1 case. A diagnosis of osteoporosis was established if any measured BMD value at a specific site fell below the normal range. BMD = bone mineral density.

This experiment was approved by the Ethics Committee of Cangzhou Hospital of Integrated Traditional Chinese and Western Medicine, Hebei Province, and the ethical approval letter number is 2019047. A total of 155 patients underwent hybrid UKAs performed by the same surgeon at our institution between January 2016 and December 2017. Of these, 150 UKAs (150 patients) with a minimum follow-up of 3 years were included in this study. Five patients were excluded due to incomplete follow-up data or protocol deviation. The mean follow-up period was 3.6 years (range, 3–6 years). The BMD of the patients was normal or decreased, but not osteoporotic (Figs. 1 and 2). There were 64 males and 91 females, with a mean age of 62.03 years (range, 47–83 years; Table 1).

**Table 1:** Demographic data.

	Oxford III hybrid (n = 150)	Loosened patients (n = 5)	*P*-value
Age (yr)	62.03 ± 8.25	64.20 ± 6.06	.779
Gender			
Male (case)	64	3	
Female (case)	86	2	
Height (m)	1.65 ± 0.75	1.66 ± 0.09	.926
BMI (kg/m^2^)	26.32 ± 3.52	26.96 ± 2.96	.727
Diagnosis			
Osteoarthritis (case)	144	5	
Osteonecrosis (case)	5	0	
Traumatic arthritis (case)	1	0	
Side			1.000
Right (case)	80	3	
Left (case)	70	2	
Comorbidity			
Coronary heart disease (case)	12	1	
Diabetes mellitus (case)	19	2	.136
Cerebral infarction (case)	11	1	
Others (case)	14	0	
Anesthetic mode			
Spinal analgesia (case)	77	3	
General anesthesia (case)	73	2	
ASA			1.000
I (case)	65	2	
II (case)	82	3	
III (case)	3	0	

There was no statistically significant difference in baseline characteristics between patients with normal follow-up and those who underwent revision surgery.

A total of 5 patients experienced prosthesis loosening (loosened patients, n = 5), of whom only 2 underwent revision surgery (revisions, n = 2). The remaining 3 patients were managed conservatively due to comorbidities or personal preference.

*P*-values were calculated using the Mann–Whitney *U* test for continuous variables and Fisher’s exact test for categorical variables due to small sample size in the Loosened group.

BMI = body mass index, ASA = American Society of Anesthesiologists.

### 2.2. Preoperative diagnosis

The preoperative diagnosis was knee osteoarthritis, knee osteonecrosis and knee traumatic arthritis. Diagnostic criteria are now detailed based on radiographic and clinical guidelines.

### 2.3. Implant

The Oxford phase-3 (Biomet, UK, LTD) hybrid UKA, with a cemented tibial component and a cementless femoral component, was used in all knees. BMD was considered in the preoperative assessment. If patients had osteoporosis, cemented UKA was used in their knees.

### 2.4. Survivorship analysis

Survival rates, standard deviations, and confidence intervals were calculated using SPSS 23.0 (SPSS Inc., Chicago). The cumulative survival rate was calculated using the Kaplan–Meier method. The follow-up period was divided into 1 year intervals. Annual success was defined as the prosthesis remaining in situ, and failure was defined as reoperation due to deep infection, revision (defined as either replacement of prosthetic components or conversion to total knee arthroplasty [TKA]), periprosthetic fractures, bearing dislocation, or lateral meniscus injury.

### 2.5. Clinical assessment

We prepared a 12-item questionnaire for patients. Scores obtained from the 12-item knee questionnaire were recorded before the operation and at the last follow-up. The Oxford knee score (OKS) is a patient-reported outcome measure consisting of 12 multiple-choice questions that assess pain and daily activities (range: 12–60, with 12 points indicating the best knee function).^[15]^ The pain and function scores were assessed both preoperatively and at the final follow-up. Range of motion (ROM) in both extension and flexion was measured preoperatively and at the last follow-up.

### 2.6. Radiographic assessment

Anteroposterior and lateral views were obtained 1 week postoperatively, and radiographs were taken annually thereafter. We evaluated RLL around the implants and the position of the implants. RLLs were assessed on both anteroposterior and lateral views and were recorded separately for the tibial plateau prosthesis and the femoral component. The width of RLLs was measured in millimeters using digital imaging software, and their locations were categorized according to standard zones described in the Knee Society Roentgenographic Evaluation system. If RLLs could be connected by the naked eye, they were considered as 1 continuous line. Two independent observers (orthopedic surgeons) evaluated all radiographs, and interobserver reliability was assessed using Cohen’s kappa coefficient.

### 2.7. Statistical analysis

All statistical analyses were performed using SPSS 23.0. Continuous variables (OKS, ROM) were reported as mean ± standard deviation. Paired *t*-tests were performed to determine statistical significance in the differences between preoperative and last follow-up values, as the data were derived from the same subjects. A *P*-value < .05 was considered statistically significant. The survival rate was calculated using the Kaplan–Meier method.

## 3. Results

### 3.1. Survival rate analysis

**Figure 3.**
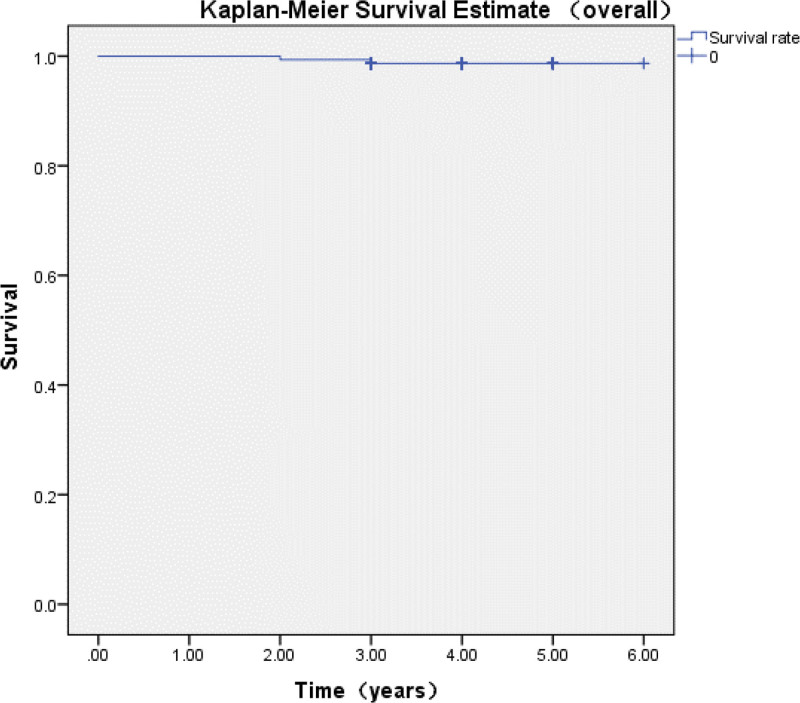
The overall survival rate at 6 years postoperatively in our study. The survival rate of the prosthesis showed a gradual annual decline, with a rate of 96.67% observed at the 6-year follow-up.

The overall survival rate at 6 years postoperatively was 98.7% (95% confidence interval [CI]: 95.0–100.0%; Fig. 3), and the overall reoperation rate was 1.3%.

### 3.2. Clinical results

**Figure 4.**
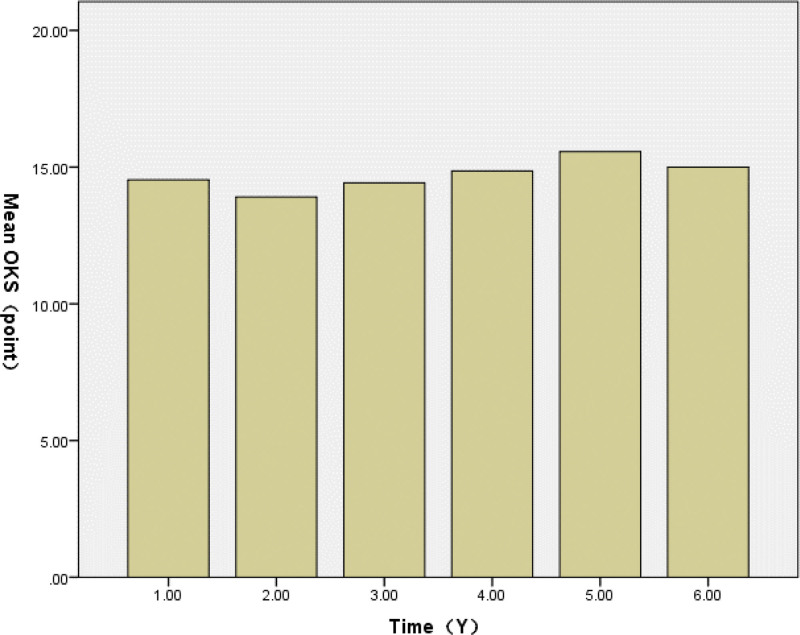
The mean OKS 1 year postoperatively to 6-year postoperatively. The Oxford knee score (OKS) showed progressive improvement and reached its optimal level at the 5-year postoperative follow-up. OKS = Oxford knee score.

**Figure 5.**
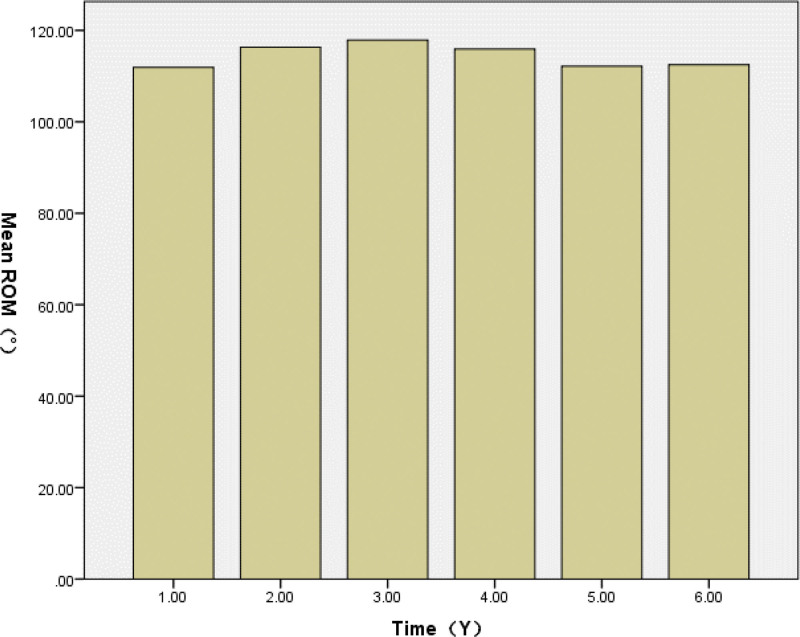
The mean ROM 1 year postoperatively to 6-year postoperatively. The range of motion (ROM) of the knee joint reached its maximum at 3 years after surgery, followed by a gradual decrease over time. ROM = range of motion.

The mean OKS improved from 40.94 (range, 29–50) preoperatively to 14.84 (range, 13–18) at the last follow-up, indicating a significant reduction in symptoms and functional improvement. The mean ROM improved from 104.81 (range, 90–130) preoperatively to 114.93 (range, 90–130) at the last follow-up (Table 2). During routine annual follow-ups postoperatively, the mean OKS and ROM varied at each follow-up. The OKS values from 1 year to 6 years postoperatively were 14.53 (range, 13–19), 13.91 (range, 12–18), 14.43 (range, 13–18), 14.86 (range, 13–18), 15.57 (range, 14–18), and 15.00 (range, 14–16; Fig. 4). The ROM values from 1 year to 6 years postoperatively were 119.92 (range, 90–130), 116.32 (range, 95–130), 117.90 (range, 100–130), 115.92 (range, 100–130), 112.17 (range, 100–125), and 112.50 (range, 110–115; Fig. 5).

**Table 2:** OKS and ROM.

Group	OKS (point)	ROM (°)
Pre-OP	40.94 ± 4.86	104.81 ± 10.03
Last follow-up	14.84 ± 1.39	114.93 ± 7.51
*P*	<.001	<.001

This table compares preoperative and postoperative knee function and range of motion (ROM); both parameters improved significantly after surgery, with statistically significant differences.

ROM = range of motion.

### 3.3. Radiographic assessment

The RLLs increased over time, reaching a total of 6 at 6 years postoperatively in 4 knees (Table 3). However, all cases were asymptomatic and showed no signs of implant loosening or clinical deterioration. Therefore, these RLLs were considered to be nonprogressive and not clinically significant. Additionally, 2 cases of lateral arthritis progression were observed 5 years postoperatively in 2 knees, and 2 more cases at 6 years in another 2 knees; however, none of these patients reported knee pain or functional limitations, and no further treatment was required.

**Table 3:** Radiolucent lines.

	Femoral radiolucent line	Tibial radiolucent line
1	0	0
2	0	2(893138, Ope-T 29/5/2019)
3	1(863142, Ope-T 13/6/2019)	2(893138, Ope-T 29/5/2019)
4	0	2(759621, Ope-T 6/7/2018)
5	0	1(664431, Ope-T 16/8/2017)
6	0	0
Total	1	5

The numbers 1 through 6 represent the follow-up years. Each year, only newly identified patients with radiolucent lines (RLLs) were marked, while those identified in previous years were not. For example, in the third year of follow-up, a newly observed RLL was found around the femoral component in patient ID 863142, and 2 RLLs were found around the tibial component in patient ID 893138.

Ope-T =operation time, RLL =radiolucent line.

### 3.4. Complications

**Figure 6.**
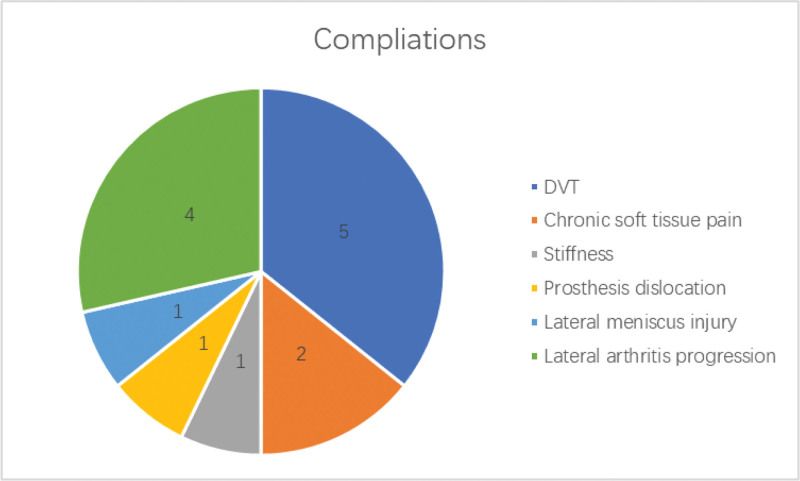
All complications after hybrid UKA within last follow-up. The most common complication was deep vein thrombosis (DVT), occurring in 5 cases, followed by progression of lateral compartment osteoarthritis in 4 cases. Other complications were less frequent, including chronic knee pain in 2 cases, and prosthetic dislocation, joint stiffness, and lateral meniscal injury in 1 case each. DVT = deep vein thrombosis, UKA = unicompartmental knee arthroplasty.

All complications are shown in Figure 6. Two knees required revision surgery. One case involved prosthesis dislocation 2 years after UKA, for which we performed a revision procedure and replaced the polyethylene insert with a thicker 1. The other case involved a meniscus injury 3 years after UKA. Initially, the patient underwent arthroscopic meniscectomy (a non-revision reoperation), but due to persistent lateral knee pain, a subsequent conversion to TKA was performed, which was classified as a revision. Other complications resolved with conservative treatment and did not require reoperation. Thus, the 6-year reoperation rate was 1.3% (n = 2), and the revision rate was also 1.3% (n = 2), as both cases ultimately underwent revision procedures.

## 4. Discussion

Several studies have reported excellent long-term survival rates for cemented and cementless UKA,^[16]^ ranging from 94% to 98% at 5 to 10 years of follow-up.^[17,18]^ In comparison, our study demonstrates a 98.7% 6-year survival rate for hybrid Oxford phase-3 UKA, which is comparable to or even slightly higher than those reported for traditional fixation methods. This suggests that hybrid fixation may offer equivalent implant longevity while potentially combining the advantages of both cemented and cementless techniques.

The main finding of this study is that the Oxford phase-3 hybrid UKA has an excellent survival rate of 98.7% at 6 years of follow-up. Several studies^[1,19-21]^ report that Oxford UKA has good long-term outcomes and a high survival rate. In our study, we confirmed that the Oxford phase-3 hybrid UKA has good short-term outcomes and survival rates. The reasons for reoperation in our study differ from the main causes reported in some literatures. Although trauma-related complications such as meniscus injury and bearing dislocation are uncommon in the early postoperative period, they may still occur due to inadequate postoperative care. In our study, the 2 complications requiring revision were both trauma-induced and associated with excessive postoperative activity or insufficient joint protection. This highlights the importance of reinforcing rehabilitation education and clearly communicating the physical limitations and care requirements following hybrid UKA, especially for active older patients.^[22]^ Studies^[23,24]^ suggest that the primary reasons for revision are progression of arthritis in other compartments, implant loosening, pain, infection, dislocation, and periprosthetic fractures. In contrast, the reasons for revision in our study were bearing dislocation and lateral meniscus injury, both of which were caused by trauma. One case involved a lateral meniscus injury diagnosed by MRI, followed by arthroscopic partial meniscectomy. However, 1 month later, the patient continued to complain of pain in the lateral knee, leading us to perform a TKA. The pain was relieved after TKA. We identified that the patient lacked sufficient awareness of protecting the reconstructed joint. Before surgery, we overemphasized the advantages of a unicondylar knee, such as its minimally invasive nature, fewer complications, and faster recovery compared to TKA. This communication led the patient to believe that their reconstructed knee would return to normal activity levels. Consequently, they were unaware of the need to protect their reconstructed knee joint, which resulted in meniscus dislocation and injury. The complications in our study showed differences between short-term and long-term follow-up outcomes.

The 1.33% revision rate in our study is a key clinical finding. This low revision rate suggests that hybrid UKA offers favorable long-term outcomes, similar to traditional UKA techniques, and may be a viable option for appropriately selected patients. Clinically, this revision rate may inform surgical decision-making, particularly when advising patients who may be considering hybrid UKA. Surgeons may take this rate into account when discussing the durability and potential complications of hybrid UKA with patients, helping them make more informed decisions. While hybrid UKA appears to provide a durable solution, individual factors such as age, activity level, and comorbidities should still guide the decision-making process.

It is challenging for surgeons to choose between UKA and TKA when the indications are unclear, even after fully evaluating the advantages and disadvantages of both procedures.^[25-29]^ The mean age of patients in our study is similar to that reported in other studies.^[30,31]^ Surgeons have performed UKA in different populations and reported the outcomes. Moore et al found that UKA in patients aged ≥80 years was associated with a higher risk of mortality, other complications, and readmission compared to patients aged <80 years.^[32]^ However, Iacono Francesco et al believed that with proper indications and accurate technique, complications and morbidity were reduced, and excellent survivorship was achieved when UKA was used in very elderly patients.^[33]^ Kim Yeong-Joon et al found that younger patients provided good clinical results and survival rates in the mid-term follow-up.^[34]^ We agree with the idea that surgeons should not regard age as a contraindication for UKA.^[35]^ We prioritized height and BMD over activity level because these factors have a more direct mechanical and structural influence on implant fit and early fixation stability, especially in cementless or hybrid fixation.^[36]^ Shorter tibial dimensions increase the risk of cortical breach and periprosthetic fracture, while insufficient BMD may compromise the osseointegration required for long-term fixation. While activity level is relevant, it is often modifiable postoperatively through education, whereas height and bone quality are innate anatomical characteristics that must be addressed preoperatively. We pay close attention to height and BMD. Shorter height means a smaller tibial plateau. The average height of patients in our study was 1.65 ± 0.75 m. Shorter patients were more likely to receive smaller sizes of tibial plateau prostheses. The rate of tibial plateau fractures was higher in patients who underwent cementless UKA. We confirmed that the patients did not suffer from osteoporosis using a bone density detector before UKA. Osteoporosis, poor alignment of the prosthesis, and small platform prostheses are risk factors for revision in UKA.^[37-39]^ The BMD of the femoral condyle is crucial for the initial stability of the prosthesis. Measurement of BMD is typically performed at the lumbar spine and both hips, as we cannot directly measure the BMD of the femoral condyle. The BMD of the spine represents the BMD of the entire body.^[32]^ This is one of the reasons why we measure BMD. Age is negatively correlated with osteoporosis.^[40,41]^ We should pay attention to factors other than just young age. The 2 cases that required reoperation were older than the average age, and both had more active lifestyles than the others.

Compared to the preoperative OKS, the knee joint function postoperatively was better. The knee joint function improved year by year during the first 2 years postoperatively and then stabilized. The ROM postoperatively was also better than preoperatively. It improved year by year during the first 3 years and then stabilized. The ROM postoperatively was also better than preoperatively. It improved year by year during the first 3 years and then stabilized.

However, a slight decline or fluctuation in OKS and ROM was observed after the third postoperative year. This may be due to age-related functional decline, reduced intensity of rehabilitation efforts over time, or degenerative changes in other compartments of the knee. These factors could contribute to the plateau or minor decrease in clinical scores, even in the absence of mechanical failure. A previous report^[42]^ indicated a high incidence of RLLs around the femoral component. There were more RLLs around the tibial plateau than around the femoral component. It is important to distinguish between physiological and pathological RLLs. However, there was still a lack of significance in predicting or indicating component loosening. We monitored the RLLs and did not provide further treatment for patients as they showed no symptoms.

The complications in our study were similar to the main complications seen with cemented or cementless UKA. Despite the specific combination mode and short-term follow-up, there was no significant difference in complications between the 3 fixation methods. The Oxford phase-3 hybrid UKA for knee anteromedial arthritis has promising outcomes at a minimum 3-year follow-up and has a survival rate of 98.7% 6 years postoperatively.

## 5. Limitations

This was a retrospective study with limited data and a relatively small sample size. The follow-up loss rate reached 3.23% (5/155), which is relatively high. We did not investigate or record patient satisfaction, and satisfaction is not necessarily positively correlated with joint function. Although patients achieved good joint function, it remains unknown whether they had high satisfaction with hybrid UKA. Moreover, selection bias is an inherent limitation, as patients were selected based on clinical indications and surgeon discretion at a single institution, potentially limiting the generalizability of the results. Additionally, confounding variables such as comorbidities (e.g., diabetes, hypertension, osteoporosis), for which data were insufficient, may influence implant survival, bone quality, or postoperative recovery were not fully controlled for or statistically adjusted. Future prospective studies with larger cohorts and multivariate analyses are needed to clarify the impact of these factors.

## Author contributions

**Data curation:** Tingting Zhou, Qingpeng Fang, Pengpeng Wang.

**Resources:** Xiaoming Li.

**Writing – original draft:** Yunchao Zhao, Yijie Yang.

**Writing – review & editing:** Jianyong Zhao.
